# Case Report: Sustained biochemical remission following early initiation of odevixibat in an infant with monoallelic ABCB11 mutation and histologically confirmed PFIC2

**DOI:** 10.3389/fped.2025.1648663

**Published:** 2025-11-27

**Authors:** Thomas Kehler, Jan Thomas Schaefer, Katja Evert, Maren Zapke, Joachim Woelfle, Michael Melter, André Hoerning

**Affiliations:** 1 University Children’s Hospital Regensburg (KUNO), University Hospital Regensburg, Regensburg, Germany; 2 Department for Pediatric Gastroenterology, Hepatology and Endoscopy, Clinic for Pediatrics and Adolescent Medicine, Friedrich-Alexander University of Erlangen-Nuremberg, Erlangen, Germany; 3 Institute of Pathology, University of Regensburg, Regensburg, Germany; 4 Clinic for Pediatric and Adolescent Medicine, Friedrich-Alexander University of Erlangen-Nuremberg, Erlangen, Germany

**Keywords:** case report, intrahepatic cholestasis, PFIC2, ABCB11, odevixibat, IBAT inhibitor

## Abstract

**Introduction:**

Progressive familial intrahepatic cholestasis (PFIC) are rare genetic hepatocellular disorders that affect bile secretion and predominantly manifest in early childhood. PFIC2, which is caused by mutations in the ABCB11 gene, often progresses to end-stage liver disease.

**Case presentation:**

We present the case of a male infant with PFIC2, which was associated with a heterozygous frameshift mutation in the ABCB11 gene as well as an additional heterozygous variant in the ATP8B1 gene. Initial clinical management involved ursodeoxycholic acid (UDCA) administration and fat-soluble vitamin supplementation. However, it was only after switching treatment to the ileal bile acid transporter (IBAT) inhibitor odevixibat at three months of age, that the patient exhibited significant improvement, including normalization of cholestasis parameters and liver enzymes. Liver function has remained stable on therapy for 2.5 years during which time the patient has maintained normal growth and development with no evidence of disease progression.

**Discussion:**

This case study highlights the effectiveness of odevixibat in managing PFIC2, demonstrating sustained disease suppression and symptomatic relief. It also emphasizes the importance of comprehensive clinical evaluation and accurate disease characterization as well as the potential of targeted therapies in improving outcomes for patients with PFIC2.

## Introduction

Progressive familial intrahepatic cholestasis (PFIC) is a heterogeneous group of rare genetic hepatocellular disorders that usually present in early childhood with cholestatic icterus. Variants in different genes cause a disorder of bile secretion (1, 2). The spectrum of PFIC subtypes continues to expand as new genetic variants are identified. The cumulative incidence of the three most common forms (types 1–3) being estimated to be 1:50,000–1:100,000 (3). Manifestations and severity of PFIC are determined by the loss of function of liver transport proteins caused by mutations such as of *ATP8B*, *ABCB11* and *ABCB4 in PFIC1 to 3*, resulting in reduced function of PFIC-associated protein 1 (FIC1), bile salt export pump (BSEP) and multidrug resistance protein 3 (MDR3) (1, 4). Other PFIC variants include gene mutations that cause deficiencies in tight junction protein 2 (TJP2), farnesoid receptor (FXR) or myosin 5B (MYO5B) (1).

PFIC is clinically characterized by severe pruritus due to excess serum bile acids. In addition to neonatal icterus the urine may often present intensely yellow to dark yellow. Over time, portal hypertension, ascites, biliary cirrhosis with hepatic steatosis, hepatocellular carcinoma and extrahepatic manifestations may develop (5, 6). Two thirds of patients with severe BSEP deficiency develop end-stage liver disease requiring liver transplantation in childhood (1). The diagnosis is based on history, clinical signs, liver ultrasonography, cholangiography, liver histology and specific laboratory tests, as well as on molecular genetic testing to confirm diagnosis (7).

There is no causal treatment for PFIC. Severe cholestatic pruritus is usually refractory to treatment with ursodeoxycholic acid (UDCA), rifampicin, cholestyramine and naltrexone (1). Targeted drug options are ileal bile acid transporter (IBAT) inhibitors odevixibat and maralixibat that reduce reabsorption of bile acids from the intestine and promote their excretion via the colon. As a result, the total amount of bile acids in the body and pruritus are reduced (8, 9). Supportive therapies include administration of fat-soluble vitamins and nutritional optimization with a high-calorie diet (10). Surgical options consist of partial biliary diversion, which provides relief of cholestatic symptoms in some PFIC1 or 2 patients, and liver transplantation (1).

Here we present a case of a male child with PFIC2 and heterozygous mutations in the *ABCB11* and *ATP8B1* genes in whom early IBAT inhibitor treatment resulted in sustained clinical and biochemical remission.

## Case report

A male newborn presented with prolonged jaundice. The stool was never found to be acholic. The attending pediatrician repeatedly measured the total bilirubin, with a decreasing tendency. At the age of 6 weeks, direct hyperbilirubinemia was detected for the first time prior to the initiation of propranolol therapy for a cutaneous haemangioma. Additionally, markedly elevated transaminases were noted, while gamma-glutamyl transferase (GGT) was not increased. Treatment with UDCA was initiated.

**Figure 1:**
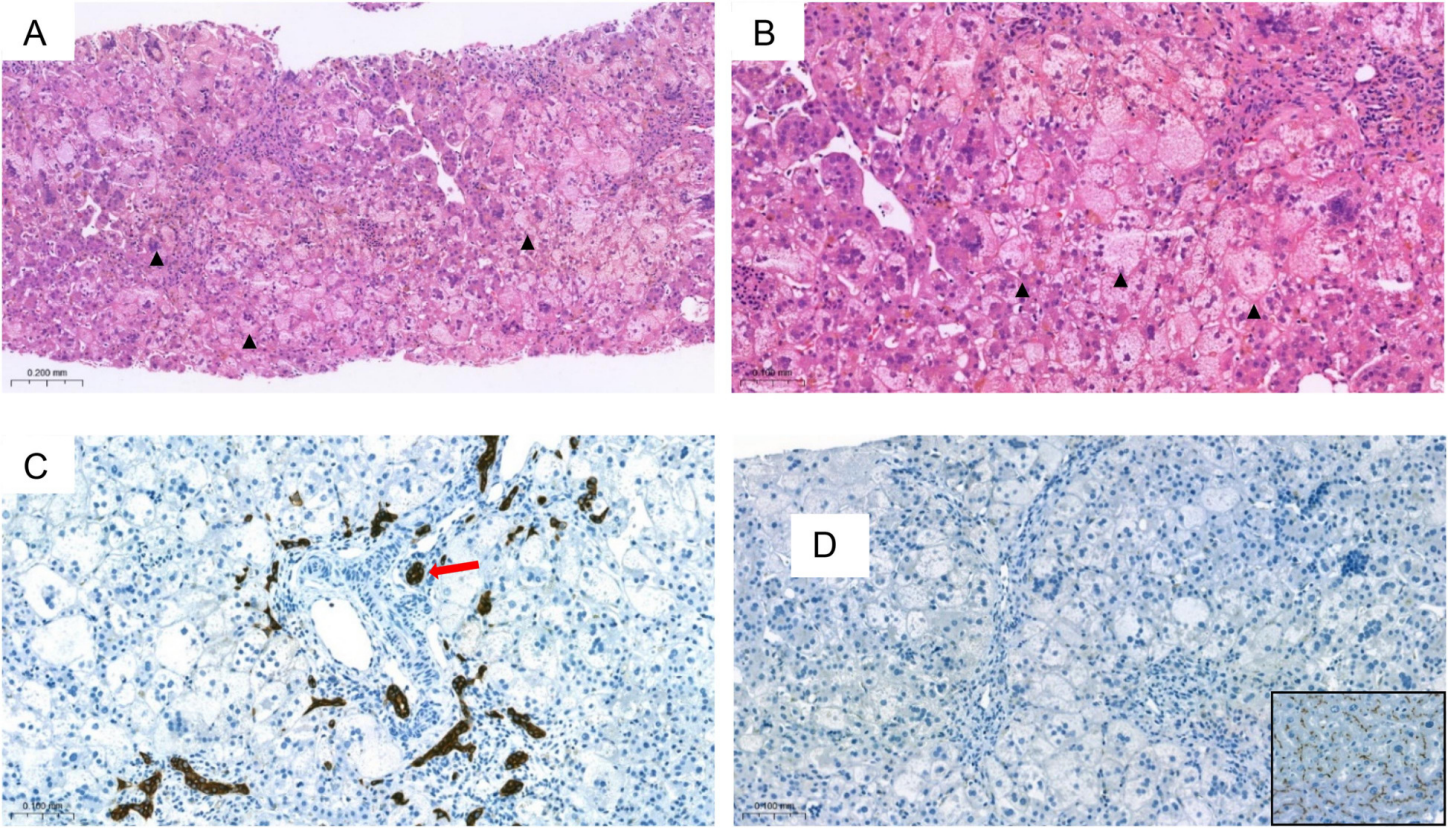
Liver histology: **(A,B)** (HE staining) showing severe cholestasis in the form of pronounced bilirubinostasis with swollen hepatocellular giant cells (arrowheads) and canalicular bilirubinostasis. In **(C)** (Cytokeratin 7 immunostaining) portal field structures can be recognized, with at least some partially recognizable regular bile ducts (arrow). There is a loss of BSEP [**(D)**, BSEP immunostaining] with positive staining of the on-slide control (Inlay 1D). BSEP, bile salt export pump; HE, hematoxylin and eosin. The immunohistochemical staining for BSEP was performed automatically (BenchMark ULTRA IHC) with on-slide controls at the accredited Institute of Pathology at the University of Regensburg.

For further evaluation of neonatal cholestasis, the patient was referred to our center at the age of 9 weeks. Apart from jaundice no other abnormalities were observed on physical examination. Abdominal ultrasound showed nonspecific findings, including hepatomegaly and increased liver echogenicity. However, the stool now appeared acholic. Endoscopic retrograde cholangiography (ERC) revealed normal extrahepatic bile ducts, effectively ruling out biliary atresia. Liver biopsy was performed at the same time, a representative histology displaying 22 portal fields could be examined. Histological examination revealed cholestatic giant cell transformation with marked cholestasis and beginning of septal fibrosis (Ishak fibrosis score 2–3/6; Figures 1A,B). Regular bile ducts can be seen in part of the portal fields (Figure 1C). Immunohistochemistry demonstrated a distinct absence of BSEP expression (Figure 1D).

**Figure 2:**
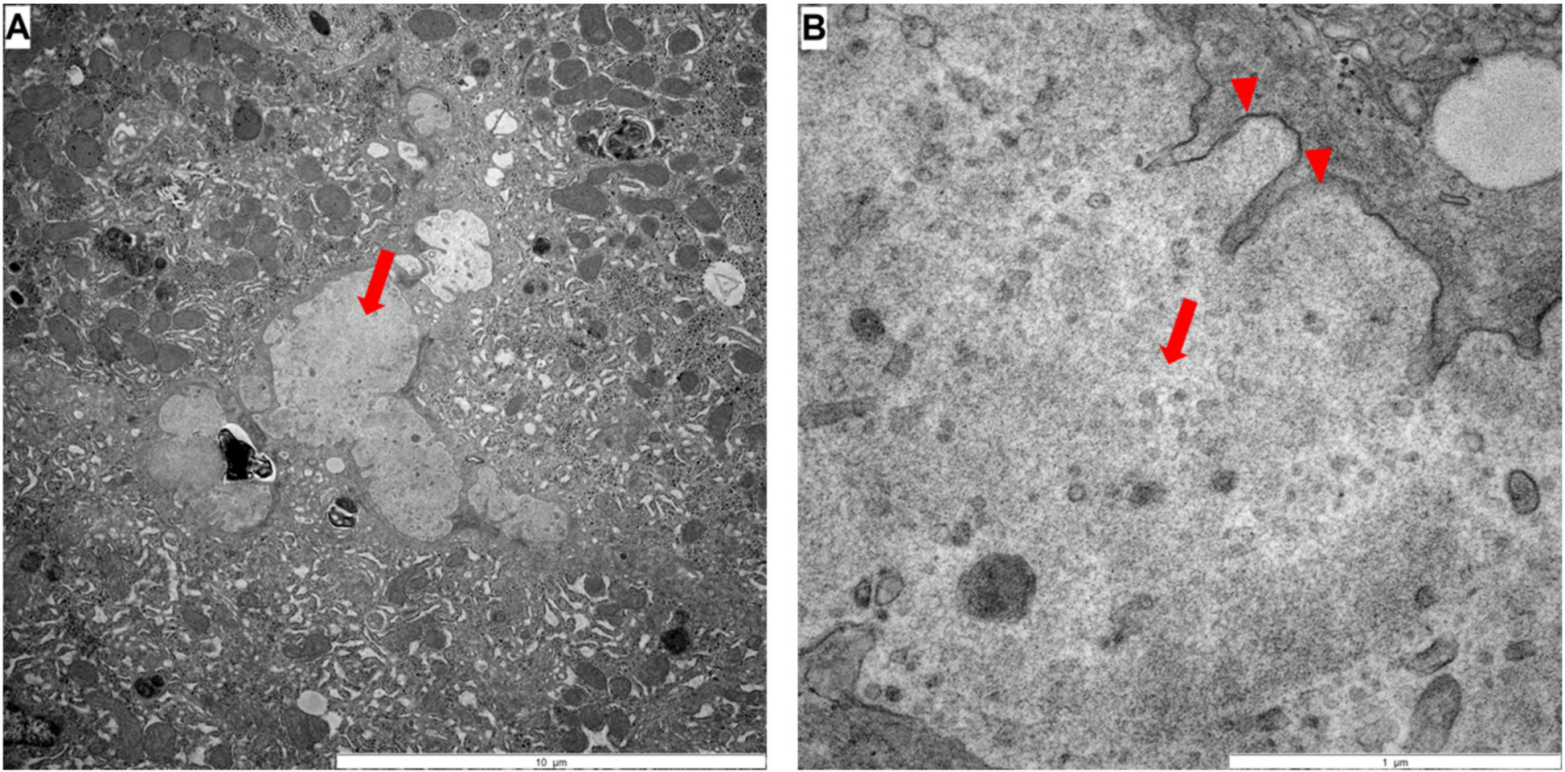
Electron microscopy: **(A,B)** large amounts of bile pigment could be found in hepatocytes, macrophages, sinusoids and occasionally in bile ducts, whereby the sinusoids are partly full of amorphous bile pigment (arrow in **A** and **B**). Residues of microvilli can be seen (arrowhead in **B**).

Electron microscopy showed no evidence of Byler's bile (Figure 2), but amorphous bile. Treatment with fat-soluble vitamins was then started. Serum bile acids were measured for the first time three weeks after initiating UDCA therapy. Despite an increased UDCA fraction, primary and total bile acids were markedly elevated.

Genetic testing including Trio-Whole-exome-sequencing revealed the presence of a likely pathogenic variant in the ABCB11 gene (c.1966_1967del, p.Leu656Alafs*9, heterozygous paternal) and a variant of uncertain significance in the ATP8B1 gene [c.208G>A, p.(Asp70Asn), heterozygous maternal]. The ABCB11 variant causes a frameshift as coding effect. No second mutation was found in the ABCB11 gene.

Since there was no indication for liver transplantation evaluation, further treatment was continued at the nearest pediatric hepatology unit. Early diagnosis and already moderate fibrosis with the risk of progressive liver damage led to an indication for treatment with an IBAT inhibitor. Odevixibat is approved for the treatment of pruritus in children with PFIC from 3 months of age in the USA. Therefore, treatment with odevixibat was started according to the prescribing information at a dose of 40 µg/kg OD from the third month onward.

**Figure 3:**
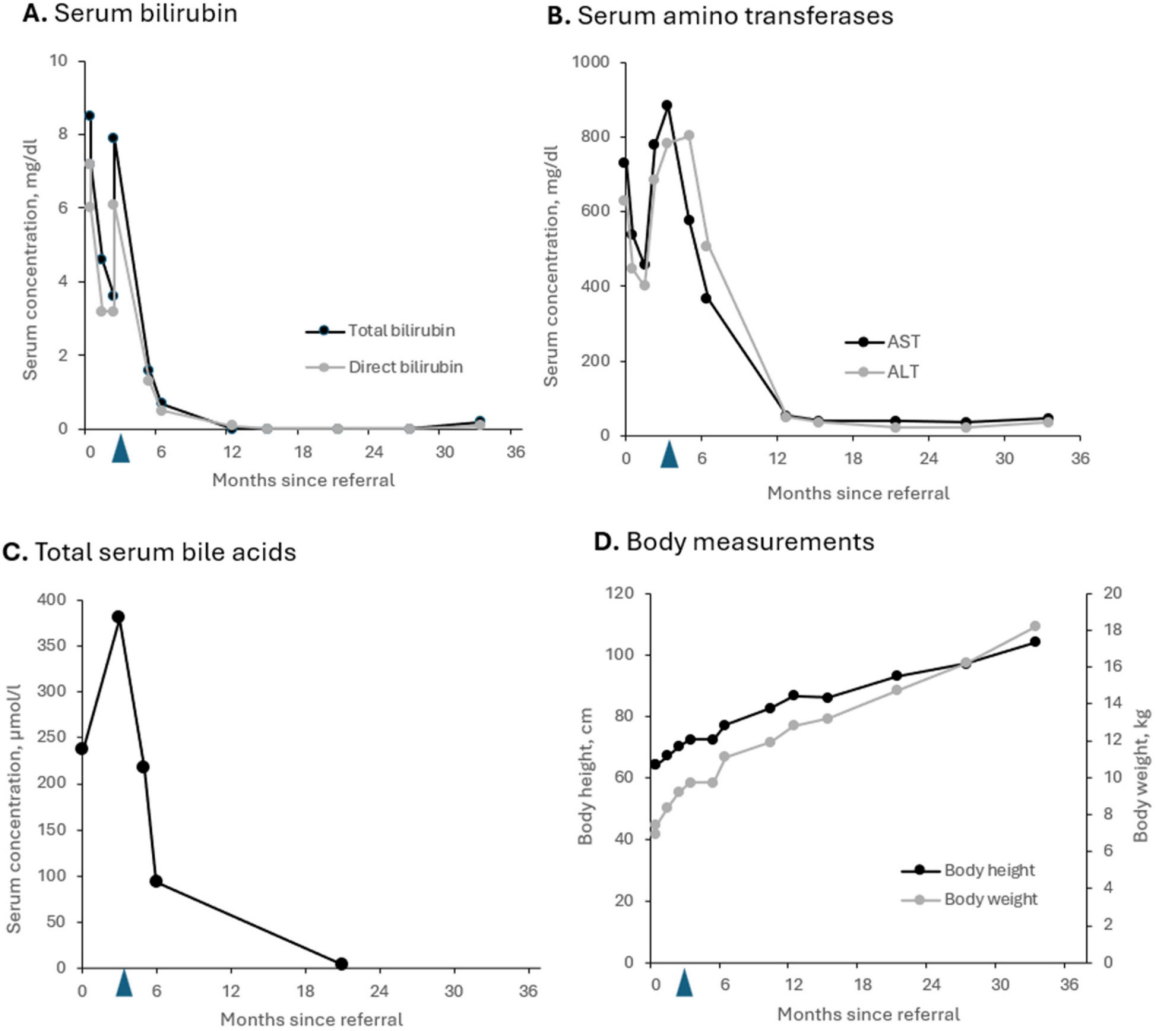
Parameters for cholestasis **(A,C)** and hepatocellular injury **(B)** as well as growth **(D)** of the patient with PFIC2 during odevixibat treatment over 2.5 years. **(A)** Total and direct bilirubin levels; **(B)** serum concentrations of amino transferases AST and ALT; **(C)** total serum bile acid levels; **(D)** body height and body weight. Blue arrows: start of odevixibat treatment at 3 months. ALT, alanine amino transferase, AST, aspartate amino transferase, PFIC, progressive familial intrahepatic cholestasis.

The aim of IBAT inhibitor treatment was to interrupt enterohepatic circulation of bile acids and thus prevent both pruritus and disease progression. The patient responded very well to odevixibat and shortly after starting the IBAT inhibitor, UDCA treatment could be discontinued. Following initiation of treatment liver function parameters including serum bile acid concentrations decreased to normal ranges within few weeks (Figure 3). Besides a transient increase in AST shortly after initiation of IBAT inhibitor therapy (Figure 3B), the patient did not experience any relevant side effects.

**Figure 4:**
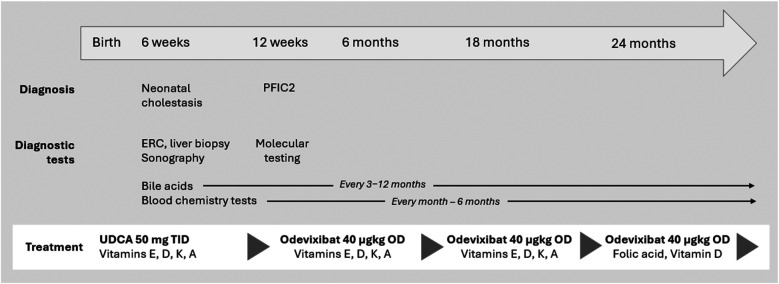
Timeline and key clinical and laboratory findings in a patient with PFIC2. PFIC, progressive familial intrahepatic cholestasis; ERC, endoscopic retrograde cholangiography; OD, once daily; TID, three times a day; UDCA, ursodeoxycholic acid.

To date, no clinical progression has been observed under treatment with odevixibat. During up to now 2.5 years of treatment serum parameters of both, cholestasis and liver injury remained constantly normal. Follow-up ultrasound examinations revealed no evidence of increasing or clinically significant liver fibrosis or portal hypertension with a portal vein peak flow of 37 cm/sec, a triphasic flow profile in hepatic veins and a spleen of normal size at the age of 3 yrs. No liver fibrosis was detected on the ARFI (Acoustic Radiation Force Impulse) assessment. The patient continues to be treated with odevixibat at a dose of 40 µg/kg/day. All co-medication has been stopped. He only has been prescribed folic acid and vitamin D supplementation (1,000 IU/day) during the winter season (Figure 4). At the last visit the patient was symptom-free; in particular, he did not report any itching. The patient was in good physical and nutritional condition, physical examination was unremarkable, and he had no jaundice. He had reached a height of 104 cm (98th percentile), body weight of 18.2 kg (96th percentile), and body mass index 16.8 kg/m^2^ (79th percentile). Taken together, he continues to be clinically indistinguishable from healthy children of the same age and no adverse events under medication occurred.

## Discussion

Firstly, this case highlights the importance of determining direct bilirubin in prolonged neonatal jaundice, as recommended by current guidelines (11). Early detection and differentiation of the total bilirubin would have accelerated the diagnosis even more. In neonatal cholestasis, there is always a risk of bleeding due to vitamin K deficiency. Due to the age of 6 weeks, direct hyperbilirubinemia and acholic stool as indicators for, e.g., biliary atresia as the most frequent and urgent cause of neonatal cholestasis had to be ruled out promptly (12, 13). Fortunately, this could be done less invasively using ERC. Because of the low GGT cholestasis, liver biopsy or/and genetic testing would probably have been sufficient if it had been suspected earlier. In this case liver biopsy was performed during the same procedure when ERC was performed. Based on histology, including immunohistochemistry and electron microscopy, PFIC type 2 was still diagnosed at an early stage. Treatment with the IBAT inhibitor odevixibat was particularly successful, resulting in complete normalization of parameters of cholestasis and liver injury.

Our patient was found to have two variants associated with intrahepatic cholestasis: a likely pathogenic variant (ACMG class 4) in the *ABCB11* gene, linked to PFIC2, and a variant of uncertain significance (ACMG class 3) in the *ATP8B1* gene, which is associated with PFIC1. Each parent carried one of the gene variants and both parents were healthy with no previous history of cholestasis. The ABCB11 variant (c.1966_1967del, p.Leu656Alafs*9) causes a frameshift leading to a premature stop codon. This mutation was reported as a predicted protein truncating mutation (14). The same mutation was documented in one compound heterozygous patient from the PEDFIC1 trial and was not included in the list of ABCB11 mutations predicting complete absence of the BSEP protein, which served as exclusion criteria for PEDFIC1 (8). But only patients with two mutations in ABCB11 were excluded and all excluded patients in that list had homozygous mutations. In a recent real-life study, 2 out of 11 patients with PFIC2 and a protein truncating ABCB11 mutation did not respond to the treatment with odevixibat (15). Both patients carried homozygous mutations. In our patient, only a single probably truncating mutation in the ABCB11 was detected. While biallelic mutations are typically required to confirm the diagnosis of PFIC2, it is conceivable that an undetected second variant, such as a deep intronic or regulatory mutation, may be present and allows for residual BSEP activity. Felzen et al. emphasized that even a single truncating mutation can negatively affect disease severity, particularly in compound heterozygous constellations (14). However, if the second allele harbors a milder variant that permits partial BSEP function, a clinical response to IBAT inhibitors may still be possible.

Despite the absence of a second mutation typically expected in autosomal recessive inheritance, the diagnosis of PFIC2 still could be established based on the absent BSEP expression in immunohistochemistry and characteristic histological features. Electron microscopy showed amorphous bile, typical of PFIC2, but no coarse granular “Byler's” bile, which can be observed in PFIC1 (16, 17). Furthermore, standard histology revealed giant hepatocytes, bile duct proliferation, and marked cholestasis, which are common histopathological features of PFIC2 and often observed at a disease stage early in life. Despite the inconclusive molecular genetic findings, liver histology appeared to provide a clear diagnosis. A likely explanation is the presence of a second, undetected variant, which in this case would result in compound heterozygosity. To elucidate this aspect further in depth a whole genome analysis is currently under way.

Mutations in the *ATP8B1*, *ABCB11* or *ABCB4* genes are associated with a continuum of cholestatic disorders. These include PFIC, benign recurrent intrahepatic cholestasis (BRIC), intrahepatic cholestasis of pregnancy (ICP) and low-phospholipid associated cholelithiasis (LPAC). BRIC was considered benign due to its late onset with recurrent episodes of cholestasis and no progression to liver fibrosis. BRIC is now often thought to be a form of episodic PFIC.

In our patient a second mutation in the *ABCB11* gene was most likely not identified. These is often seen in case series revealing the underlying mutations (16, 18). The time course and the progressive liver fibrosis makes the diagnosis of BRIC unlikely. Still a modifying effect of the *ATP8B1* mutation could not be excluded. The presence of a predicted protein truncating mutation generally leads to a more severe disease course. This could not be observed in our patient.

Our findings of a significant reduction of bile acid concentrations as well as of cholestasis and liver injury parameters, together with resolution of pruritus by odevixibat are consistent with previous case reports (19–22) and the results of the phase 3 clinical trial (8) including the results from an open-label long-term study (23) in patients with various types of PFIC. In our patient with a histological BSEP deficiency, treatment with the IBAT inhibitor resulted in sustained clinical and biochemical remission.

In conclusion, this report highlights the importance of an early and accurate diagnosis and emphasizes the value of targeted IBAT inhibitor therapy in improving biochemical and clinical outcomes in PFIC2 patients.

## Patient perspective

From a patient's viewpoint, odevixibat treatment may significantly improve quality of life by reducing severe pruritus and preventing disease progression. Early diagnosis and personalized therapy lead to better symptom management and possibly avoidance of invasive procedures.

## Data Availability

The raw data supporting the conclusions of this article will be made available by the authors, without undue reservation.
